# Beliefs, barriers, and promotion practices of Indian nurses’ regarding healthy eating for cancer survivors in a tertiary care hospital—A cross sectional survey

**DOI:** 10.7717/peerj.17107

**Published:** 2024-03-19

**Authors:** Hritika D. Pai, Stephen Rajan Samuel, K. Vijaya Kumar, Charu Eapen, Alicia Olsen, Justin W.L. Keogh

**Affiliations:** 1 Department of Physiotherapy, Kasturba Medical College Mangalore, Manipal Academy of Higher Education, Manipal, Karnataka, India; 2 Division of Supportive Care in Cancer, Department of Surgery, University of Rochester, Rochester, NY, United States of America; 3 Department of Paediatrics, Stanford University School of Medicine, Stanford, CA, United States of America; 4 Faculty of Health Sciences and Medicine, Bond University, Gold Coast, Australia; 5 Human Potential Centre, Auckland University of Technology, Auckland, New Zealand

**Keywords:** Cancer, Neoplasm, Nurses, Nutrition, Survivorship

## Abstract

**Purpose:**

To describe the beliefs, barriers and promotion practices of Indian nurses’ regarding healthy eating (HE) behaviours amongst cancer survivors, and to gain insights into whether their educational qualifications might affect the promotion of HE.

**Methods:**

Data was gathered using a validated questionnaire, 388 of the approached 400 nurses who worked at a tertiary care hospital in India gave informed consent to participate in the study. The Mann-Whitney U test and the Chi square analysis (for continuous and categorical variables respectively) were performed to carry out sub-group comparisons based on the qualification of the nurses i.e., Bachelor of Science in Nursing (BSc) and General Nursing and Midwifery (GNM).

**Results:**

The nurses believed that dieticians/nutritionists were primarily responsible for educating the cancer survivors regarding HE. HE was promoted by nurses’ relatively equally across multiple treatment stages (“during” treatment 24.4%, “post” treatment 23.1%; and “pre” treatment 22.3%). Nurses’ believed HE practices had numerous benefits, with improved health-related quality of life (HRQoL) (75.7%), and mental health (73.9%) being the most frequent responses. The most frequently cited barriers by the nurses in promoting HE were lack of time (22.2%), and lack of adequate support structure (19.9%). Sub-group comparisons generally revealed no significant difference between the BSc and GNM nurses in their perceptions regarding HE promotion to cancer survivors. Exceptions were how the GNM group had significantly greater beliefs regarding whether HE can “reduce risk of cancer occurrence” (*p* = 0.004) and “whether or not I promote HE is entirely up to me” (*p* = 0.002).

**Conclusion:**

The nurses in India believe in the promotion of HE practices among cancer survivors across various stages of cancer treatments. However, they do face a range of barriers in their attempt to promote HE. Overcoming these barriers might facilitate effective promotion of HE among cancer survivors and help improve survivorship outcomes.

**Implications for cancer survivors:**

Indian nurses employed in the two tertiary care hospitals wish to promote HE among cancer survivors, but require further knowledge and support services for more effective promotion of HE.

## Introduction

In the year 2020, there were over 19.3 million incident cases of cancer across the globe, according to the Global Cancer Observatory (GLOBOCAN), with India ranked third after China and the USA (Sung et al., 2021; World Health Organization, 2024). Interestingly, the amount of peer-reviewed published research on cancer in India and China is remarkably low, meaning the external validity of the research conducted in other parts of the world may not necessarily apply.

Primarily, cancer is characterized by an uncontrolled genetic cell growth and proliferation. However, environmental and lifestyle changes are attributable factors for the onset of the disease, *e.g.*, obesity, diet, tobacco and alcohol consumption (Bingham & Riboli, 2004; Anand et al., 2008; Morze et al., 2021). The recent advancements in anti-cancer treatments in countries such as India for *e.g.*, chemotherapy, radiotherapy, hormonal therapies, and multimodal treatment approaches have caused a drastic increase in survival rates post-diagnosis (Singh et al., 2018). Unfortunately, many of these cancer-related treatments severely affect the survivors quality of life (QoL) as well as their physical and mental well-being (Mazzotti et al., 2012).

Cancer related treatments (hormonal therapies) lead to cachexic and sarcopenic obesity which are characterized by significant loss of muscle and bone mass and increase in fat mass, respectively (van Dillen et al., 2014). Specifically, chemotherapy and hormonal therapies, *e.g.*, androgen deprivation therapy in prostate cancer, lead to major changes in the body composition of the cancer survivors that can put them at a risk of cachexic or sarcopenic obesity (Oefelein Michael et al., 2002; Galvão et al., 2008; Kintzel et al., 2008; Hadji et al., 2012; Young et al., 2014). These changes can result in the patients being susceptible to developing co-morbidities such as cardiometabolic syndromes, osteoporosis, and increased risk of fractures, which in turn result in lower survival rates (Sparano et al., 2012).

There is evidence to support the belief that improvements in dietary choices can offset some of these cancer treatment related comorbidities, leading to enhanced quality of life and health related outcomes thus resulting in reductions in levels of obesity, diabetes, as well as cancer recurrence (Mokdad et al., 2003; Millar & Davison, 2012; Langius et al., 2013). Research suggests that when health care professionals involved in the oncology care team *e.g.*, oncologists, physical therapists, and/or nurses frequently promote lifestyle modifications among cancer survivors, they are more likely to increase their healthy behaviors. However, the promotion of these healthy behaviors is often beyond their scope of practice or the health care professionals core field of expertise (Jones & Demark-Wahnefried, 2006; Karvinen et al., 2012; Puhringer et al., 2015). Hence, there is a need to establish a referral pathway that better provides cancer survivors with evidence-based recommendations regarding healthy behaviors such as dietary patterns.

Nurses play a crucial role in fostering communication between cancer survivors and the larger oncology care team, as they interact with their patients more frequently than the rest of the oncology care team and they are also able to spend greater amounts of time, during which they can counsel the survivors regarding supportive care issues (Leahy et al., 2013). Nurses may also view themselves as important health professionals for encouraging healthy eating (HE) among cancer patients (van Dillen et al., 2014; Blake & Patterson, 2015). However, there is a paucity of evidence from India regarding the promotion practices and the beliefs of the nurses regarding HE among cancer survivors. The primary goal of the current study aimed at gaining insights into the HE beliefs, promotions practices and barriers of nurses in India among cancer survivors. This is an important topic to be addressed as the estimated number of cancer cases in India in 2022, was 1,461,427 and one in nine people are likely to develop cancer in India in their lifetime (Sathishkumar et al., 2022). The secondary goal of the study was to gain a preliminary understanding into whether the two educational qualifications (BSc or GNM) pathways for nurses in India, impact their beliefs, practices, and barriers regarding HE.

## METHODS

### Study Design

This cross-sectional survey aimed to gain preliminary insights into the beliefs, barriers, and promotion practices of Indian nurses’ regarding HE among cancer survivors. The questionnaire used in this study is based on the one used among Australasian nurses’ (Puhringer et al., 2015). The questions included in this survey were in English and based on the social cognitive theory and theories of planned behavior. The questionnaire also consisted of questions (as described in a previous study) about physical activity (PA) promotion practices to minimize any potential response bias among the participants, so that nurses who promoted regular PA but not HE among cancer survivors were also likely to participate in the survey (Pai et al., 2022).

### Participants and procedures

Four hundred nursing staff from a tertiary care hospital in India were requested to participate in this study. These nurses had all completed tertiary training with either a BSc (Bachelor of Science in Nursing) or GNM (General Nursing and Midwifery) degree, as these are the two pathways approved by the Indian Nursing Council to pursue a career in nursing in India (Indian Nursing Council, 2024). For enrolling into a BSc degree (4 years) the trainee must successfully complete high school subjects in physics, chemistry, and biology prior to enrollment. The nursing staff who have successfully completed mathematics subjects, can enroll in a 3-year GNM program.

The sample size estimation was carried out using estimation of proportion method. For the present study the sampling technique incorporated was convenience sampling method. A sample size of 385 participants was determined with a 5% margin of error at a 95% confidence level while estimating the population proportion of 50% based on a pervious study conducted among Australasian nurses’ wherein 52.8% of the nurses promoted healthy eating among patients with cancer during all stages of cancer treatment (Puhringer et al., 2015). Hence based on the previous assumption the proportion of nurses in the current study was approximated to 50% in the “estimation of proportion” formula.

The study protocol and the survey questionnaire were approved by the Scientific Committee and the Institutional Ethics Committee, Kasturba Medical College, Mangalore, Approval No: IEC KMC MLR 02-2021/59. After acquiring the ethical approval, the medical superintendents and the nursing superintendents of the respective hospitals were contacted as they were the relevant gatekeepers. A meeting was set up by the nursing superintendents of the two wings in the relevant hospital wards. One of the primary outcomes of these meetings was that while online surveys have become popular to assess health promotion behaviors among health care professionals in many countries, the senior nursing staff indicated that physical copies rather than online versions of the questionnaires would be preferred by their nurses. The email addresses and the phone numbers of the investigators were listed in the informed consent forms, who could be contacted if the participants had any questions regarding the survey. The investigators followed-up with the participants on alternate days in case of any queries. Any queries regarding the survey were sorted through telephone conversations with the participants.

### Instrument

The survey questionnaire used in this study is based on the framework previously described among Australasian nurses (Puhringer et al., 2015). The questionnaire included three domains *i.e.,* nurses’ demographic characteristics, prevalence of health promotion behaviors and the motivational aspects associated with the promotion of healthy behaviors amongst cancer survivors. Questions regarding the demographic characteristics of the nurses such as their age, gender, highest professional qualification, years of practice, type of hospital (public/private), the location of their respective hospitals as well as questions pertaining to their HE habits were also included.

Several multiple-choice questions were incorporated to gain insights into the nurses’ beliefs regarding HE as well their HE promotion practices among cancer survivors *e.g.*, which group of health care professionals according to them were primarily responsible for promoting HE practices among cancer survivors. In addition, questions regarding the promotion of HE during different stages of cancer treatment *e.g.*, pre-, during or post- treatment) were assessed using multiple choice questions.

Likert scale responses were utilized to determine the strength of the nurses’ beliefs regarding HE practices among cancer survivors ranging from 1 to 4, 1–strongly disagree, 2–disagree, 3–agree, 4–strongly agree. The scale evaluated nine components pertaining to HE which are (1) improves health related quality improves of life, (2) improves weight management, (3) improves fatigue levels, (4) improves mental health, (5) improves activities of daily living, (6) reduces risk of cancer recurrence, (7) reduces risk of other chronic diseases, (8) reduces tumor specific comorbidities, (9) no benefits. The participants were also asked questions regarding whether the cancer survivors are generally uninterested in HE, and whether the promotion of HE habits is entirely up to them. Additionally, questions regarding the beliefs of their fellow nurses about the promotion of HE habits among cancer survivors and if there was a strong evidence base suggesting that they should promote HE habits among cancer survivors.

Furthermore, the most cited barriers by the nurses’ in promoting HE practices among cancer survivors were also incorporated in the survey questionnaire. The barriers included, lack of time, risk to patient, lack of adequate support structure, lack of knowledge, lack of expertise, promoting HE is not their job, they do not promote HE, and they do not face any barriers promoting HE among cancer survivors.

Since there are several differences in the cultural, language and health care systems in India and Australia, modifications were made by the authors with respect to the original questionnaire (Keogh et al., 2017). The revised questionnaire was provided to eight Indian experts (validators) from the departments of physical therapy (*n* = 5), oncology (*n* = 2), and nursing (*n* − 1). In addition, the revised questionnaires were also administered to a sample of 10 nursing staff from the tertiary care hospital for. The respondents were asked open ended questions about the items in the questionnaire and what their corresponding responses meant. The responses were marked on a 3–point scale *i.e.,* agree, neutral and disagree. Questions with maximum number of “disagree” were eliminated from the survey. Questions with the greatest number of “neutral” responses were discussed further among a panel of experts to improve question clarity or eliminate specific questions, based on the suggestions provided. Post validation questions 4, 5 and 10 were omitted from the original questionnaire due to the repetitive nature of questions 4, 5. Question 10 *i.e.,* “Please list any peer reviewed journal you have subscribed to-” was omitted from the questionnaire post validation as the nurses working in India are not frequently exposed to journal clubs and the advantages of peer reviewed journals. Further, questions 21 and 23 were reframed.

### Statistical analyses

The demographic characteristics of the nurses, promotion, practices, and beliefs of the nurses regarding HE were analyzed using descriptive statistics. The descriptive data were presented as mean and standard deviation or counts and frequencies for continuous and categorical data respectively. Subgroup analyses were conducted based on the qualification of the nursing staff in the two wings *i.e.,* Bachelor of Science in nursing (BSc Nursing) or General Nursing and Midwifery (GNM). Subgroup comparisons could not be performed for other categories such as years of practice, hospital type, and location of hospital due to unequal size in these groups. For all categorical variables, the Chi-square test of association for independent samples was conducted. For all continuous variables (non-normally distributed data) the Mann–Whitney U test was conducted. Data was analyzed using the software *Jamovi* version 1.6.23, with a *p* value of <0.05 considered statistically significant.

## Results

The two tertiary care hospitals comprised approximately 600 nurses. In accordance with the pre-set sample size, 400 nursing staff working in a tertiary care hospital were approached to participate in the study. Of the 400 nurses approached, 388 responses were obtained, resulting in a response rate of 97%. Table 1 describes the demographic characteristics of the nurses. Majority of the respondents were females (97.6%). The mean age of nursing staff was 34.4 ± 9.5 years, with 11.3 ± 9.5 years of nursing, of which 2.0 ± 3.9 was the specific number of years of practice with cancer patients. Most of the nursing staff preferred a healthy diet *i.e.,* 46.6% of the BSc nursing staff and 53.4% of the GNM nursing staff.

**Table 1:** Demographic characteristics of the nurses.

	BSc		GNM	
Characteristic	**Frequency (n)**	**Percentage (%)**	**Frequency (n)**	**Percentage** **(%)**
Age (years)	**178**	**46**.**7%**	**203**	**53**.**2%**
(*n* = 381)				
Younger than 26	49	27.5%	31	15.2%
26-35	74	41.5%	90	44.3%
36-45	34	19.1%	41	20.1%
46-55	18	10.1%	37	18.2%
56-65	3	1.6%	4	1.9%
Gender (*n* = 384)	**179**	**46**.**6%**	**205**	**53**.**4%**
Female	175	45.6%	200	52.0%
Male	4	1.0%	5	1.3%
Highest Qualification (*n* = 384)	179	46.6%	205	53.4%
(BSc and GNM)				
Years of Practice (*n* = 374)	**175**	**46**.**7%**	**199**	**53**.**2%**
Fewer than 5	67	38.2%	34	17%
5-14.9	69	39.4%	100	50.2%
15-24.9	22	12.5%	30	15%
More than equal to 25	17	9.7%	35	17.5%
Years of Practice in Tumor/Cancer Group (*n* = 384)	**179**	**46**.**6%**	**205**	**53**.**3%**
Fewer than 5	35	19.5%	51	24.8%
5-14.9	19	10.6%	20	9.7%
15-24.9	3	1.7%	4	1.9%
More than 25	0	0	2	0.9%
No experience	122	68.1%	128	62.4%
Working Hospital Type (*n* = 377)	**176**	**46**.**7%**	**201**	**53**.**3%**
Public	1	0.5%	4	1.9%
Private	175	99.4%	197	98%
Location (*n* = 381)	**176**	**46**.**2%**	**205**	**53**.**8%**
Metropolitan	33	18.7%	21	10.2%
Regional	127	72.1%	156	76%
Rural	16	9%	28	13.6%
I eat healthy on a regular basis (*n* = 378)	**176**	**46**.**6%**	**202**	**53**.**4%**
Yes	152	86.3%	167	82.6%
No	24	13.6%	35	17.3%

**Notes.**

BSc Bachelor of Science in Nursing GNM General Nursing and Midwifery

The percentages for each of the outcomes in bold for the BSc and GNM groups are expressed as a percentage of the total sample of nurses. Consequently, these percentages should add to 100%. The percentages within the BSc and GNM categories for each of the outcomes in bold are expressed as a percentage of the subgroup of nurses who responded to that question.

Details regarding the current beliefs of the nurses regarding HE practices among cancer survivors is provided in Table 2. The perceptions of the nurses *i.e.,* both BSc and GNM regarding who was the primary person responsible for promoting HE habits among cancer survivors were similar. The nurses believed that the nutritionist or dietician was the primary person responsible for promoting HE among cancer survivors (79.6%). 34.8% of the nurses promoted healthy eating during all stages of cancer treatment. In addition, 45.5% of nurses promoted healthy eating in the pre-treatment phase. Subgroup comparisons demonstrated no significant differences between BSc and GNM nurses on the primary person responsible for promoting HE among cancer survivors (*p* = 0.175) or which stages of cancer treatment was HE practices most promoted (*p* = 0.546).

**Table 2:** Nurses’ current beliefs regarding healthy eating beliefs and practices.

Variable *n* = 384(%) (Missing Data/No Response=2)	Educational Qualification		Total *n* = 382
	**BSc** *n* = 178 (46.37%)	**GNM** *n* = 204 (53.10%)	
A) In your opinion, who is the primary person responsible for promoting healthy eating to your patients with cancer?			
Me	6 (3.4)	6 (2.9)	12 (3.1)
Physiotherapist	5 (2.8)	16 (7.8)	21 (5.5)
Oncologist	23 (12.9)	15 (7.4)	38 (9.9)
Exercise Physiologist	1 (0.6)	2 (1.0)	3 (0.8)
Nutritionist/Dietician	141 (79.2)	163 (79.9)	304 (79.6)
Don’t Know	1 (0.6)	2 (1.0)	3 (0.8)
Others	1 (0.6)	0 (0)	1 (0.3)

Variable *n* = 384(%) (Missing Data/No Response=3)	**Educational qualification**		**Total** ***n* = 187**
	BSc *n* = 92 (49.2%)	GNM *n* = 95 (50.80%)	
B) Indicate the stage(s) of cancer treatment at which healthy eating is promoted to your patients with cancer.			
Pre-treatment	40 (21.3)	45 (24.0)	85 (45.5)
Pre + Post –treatment	15 (8.0)	7 (3.74)	22 (11.7)
Pre + during –treatment	1 (0.5)	1 (0.53)	2 (1.1)
Every Stage	32 (17.1)	33 (17.6)	65 (34.8)
Don’t Know	4 (2.1)	9 (4.8)	13 (7.0)

**Notes.**

Group differences are based on Chi-Square test of association for independent samples.

Multiple-choice answers were possible.

BSc Bachelor of Science in Nursing GNM General Nursing and Midwifery

Percentages are calculated based on Observed % of total value.

Table 3 summarizes the nurses’ beliefs about the benefits of heathy eating practices among cancer survivors. It was observed that majority of the nurses (based on the number of nurses who stated Agree or Strongly Agree) agreed that HE habits brought about improvements in the survivors’ health related quality of life (89.3%), mental health (88.8%), ability to perform activities of daily living (88.0%), and weight management (86.4%).

**Table 3:** Nurses’ beliefs about benefits of healthy eating practices among cancer survivors.

Beliefs	Strongly disagree		Disagree		Agree		Strongly agree	
	**n**	**%**	**n**	**%**	**n**	**%**	**n**	**%**
Improves weight management (*n* = 382)	20	5.2	32	8.4	261	68.3	69	18.1
Improves activities of daily living (*n* = 381)	25	6.6	21	5.5	270	70.9	65	17.1
Reduces risk of other chronic diseases (*n* = 379)	22	5.8	59	15.6	237	62.5	61	16.1
Improves fatigue level (*n* = 383)	20	5.2	43	11.2	260	67.9	60	15.7
Improves mental health (*n* = 376)	18	4.8	24	6.4	278	73.9	56	14.9
Reduces risk of cancer recurrence (*n* = 376)	17	4.5	65	17.3	243	64.6	51	13.6
Improves health-related quality of life (*n* = 383)	24	6.3	17	4.4	290	75.7	52	13.6
Reduces tumor specific comorbidities (*n* = 377)	30	8	75	19.9	223	59.2	49	13
No benefits (*n* = 341)	122	35.8	86	25.2	114	33.4	19	5.6
My patients with cancer are generally uninterested in healthy eating (*n* = 376)	45	12.0	115	30.6	204	54.3	12	3.2
Whether or not I promote healthy eating to my patients with cancer is entirely up to me (*n* = 374)	42	11.2	85	22.7	220	58.8	27	7.2
My fellow nurses believe I should be promoting healthy eating to my patients with cancer (*n* = 379)	25	6.6	54	14.2	254	67	46	12.1
There is a strong evidence base suggesting that I should promote healthy eating to my patients with cancer (*n* = 376)	37	9.8	49	13	247	65.7	43	11.4

Table 4 illustrates the comparisons of the two subgroups of nurses’ (BSc and GNM) regarding their beliefs about the benefits of HE among cancer survivors. These analyses indicated that the GNM group had significantly greater scores on their responses to whether HE practices can “reduce risk of cancer occurrence” (*p* = 0.004) and “whether or not I promote HE is entirely up to me” (*p* = 0.002).

**Table 4:** Comparison of the nurses’ beliefs about benefits of healthy eating practices for cancer survivors based on their educational qualifications.

Belief	Educational qualification				Between group *p* value
	**BSc**		**GNM**		
	** $\bar {X}$ **	**SD**	** $\bar {X}$ **	**SD**	
Improves weight management	3.01	0.74	2.98	0.64	0.41
Improves mental health	2.95	0.69	3.02	0.58	0.33
Improves activities of daily living	2.97	0.74	3.00	0.66	0.92
Improves health-related quality of life	3.00	0.72	2.94	0.59	0.10
Improves fatigue level	2.93	0.75	2.95	0.63	0.81
Reduces risk of other chronic diseases	2.85	0.78	2.92	0.69	0.34
Reduces risk of cancer recurrence	2.77	0.75	2.96	0.62	**0.004**
My fellow nurses believe I should be promoting healthy eating to my patients with cancer	2.82	0.77	2.87	0.65	0.69
There is a strong evidence base suggesting that I should promote healthy eating to my patients with cancer	2.74	0.84	2.83	0.70	0.44
Reduces tumor specific comorbidities	2.75	0.83	2.79	0.70	0.37
Whether or not I promote healthy eating to my patients with cancer is entirely up to me	2.49	0.83	2.74	0.70	**0.002**
My patients with cancer are generally uninterested in healthy eating	2.44	0.78	2.53	0.70	0.31
No benefits	2.02	0.96	2.14	0.94	0.20

**Notes.**

Values in bold indicate *p* < 0.05, Group differences were calculated using the Mann–Whitney U test.

All items are rated on a 4 -point Likert-type scale from 1(strongly disagree) to 4(strongly agree).

BSc Bachelor of Science in Nursing GNM General Nursing and Midwifery

The summary of the most frequently cited barriers by the nurses’ (both BSc and GNM) in promoting heathy eating practices among cancer survivors is presented in Table 5. Majority of the nurses in both groups were neutral regarding the barriers listed in the survey. The most frequently cited ones included lack of time (39.5%), expertise (36.2%), and adequate support structure (35.9%). No significant differences were observed between the two groups regarding perceived barriers to promotion of HE practices (*p* = 0.13−0.94).

**Table 5:** Most frequently cited barriers by nurses in promoting healthy eating practices among cancer survivors.

Barrier	Professional qualification						Between group *p* value	Between group *χ*^2^ value
	**BSc**			**GNM**				
	**Most likely**	**Neutral**	**Least likely**	**Most likely**	**Neutral**	**Least likely**		
	**n (%)**	**n (%)**	**n (%)**	**n (%)**	**n (%)**	**n (%)**		
Lack of expertise (*n* = 330)	59(**18.1**)	72(22.1)	24(7.4)	59(18.1)	88(27.0)	24(7.4)	0.66	0.81
Lack of time (*n* = 356)	61(**17.3**)	81(23.0)	21(6.0)	78(22.2)	91(25.9)	20(5.7)	0.68	0.76
Risk to patient (*n* = 338)	56(**16.8**)	73(21.9)	22(6.6)	60(18.0)	95(28.4)	28(8.4)	0.72	0.67
Lack of adequate support structure (*n* = 360)	57(**16.0**)	91(25.6)	21(5.9)	71(19.9)	89(25.0)	27(7.6)	0.49	1.40
Lack of Knowledge (*n* = 339)	49(14.6)	74(22.1)	26(7.8)	73(21.8)	86(25.7)	27(8.1)	0.45	1.57
Other (*n* = 205)	29(14.4)	48(23.9)	15(7.5)	36(17.9)	57(28.4)	16(8.0)	0.94	0.12
Do not promote healthy eating (*n* = 299)	36(12.2)	59(20.0)	35(11.9)	56(19.0)	80(27.1)	29(9.8)	0.13	3.99
Do not have barriers in promoting healthy eating (*n* = 289)	34(11.9)	65(22.8)	29(10.2)	45(15.8)	79(27.7)	33(11.6)	0.90	0.20
Not my job (*n* = 291)	31(10.8)	59(20.6)	42(14.6)	42(14.6)	74(25.8)	39(13.6)	0.44	1.63

**Notes.**

Group differences are based on Chi-Square test of association for independent samples.

The respondents were asked to indicate the three most likely factors that prevent them from promoting healthy eating (with 1 being the most likely and 3 being the least likely).

BSc Bachelor of Science in Nursing GNM General Nursing and Midwifery

## Discussion

This cross-sectional survey was conducted among nursing staff in a tertiary care hospital in Mangalore, Karnataka, India. The study aimed at understanding the beliefs, barriers, and promotion practices of the Indian nurses regarding HE among cancer survivors.

The results of the survey demonstrate that majority of the nurses considered a nutritionist or dietician as the primary person responsible for promoting HE among cancer survivors, followed by an oncologist. However, the study among Australasian nurses wherein the original survey was first conducted suggested that the nurses (32.5%) believed that they played an important role in providing advice regarding HE practices as well promotion of PA among cancer survivors (Puhringer et al., 2015; Keogh et al., 2017). As the number of dietitians working with cancer patients in India is very limited, such results suggest, improvement of the overall survivorship outcomes among cancer survivors in India may benefit from other healthcare professionals being more active in promoting the potential benefits of HE practices in cancer care. If this was to happen, cancer nurses would be an ideal position to better collaborate with dietitians and other healthcare professionals in improving the referral pathway for cancer survivors to access evidence-based support to improve their eating practices.

There was an even mix of the stage of cancer treatment (pre-, during, and post- treatment) for which the nurses most promoted HE to cancer survivors, with approximately one sixth of the nurses promoting HE across all stages (17.1%) of cancer treatment. Such results were somewhat surprising as it is believed that HE is essential across all stages of cancer treatment, although the primary benefits of this behavior may differ at various stages of cancer treatment. For example, cancer survivors may experience loss of appetite or nausea as a result of treatments such as chemotherapy, and their nutritional demands often vary during various phases of cancer treatment (Young et al., 2014), Hence, it is of utmost importance for cancer survivors to match their nutritional intake to their current challenges (often influenced by the stage and type of cancer treatment), so to maintain their body composition, QoL and overall health during these phases (Hung et al., 2013; Aapro et al., 2014).

The nurses believed that HE had a myriad of benefits for cancer survivors. Most of the nurses “agreed” eating healthy on a regular basis improved the survivors’ health–related QoL, mental health, performance of activities of daily living and weight management. However, 57.5% of the nurses’ believed that their patients were “uninterested” in HE. This perception might be due to the lack of incorporation HE practices among cancer survivors in the Indian health care facilities across various stages of the disease. These results indicate that there is a need to strengthen referral pathways that would incorporate the promotion of HE practices among cancer survivors by health care professionals and not be restricted to dieticians/nutritionists. There also arises a need to formally train the nursing staff in the Indian health care facilities regarding the importance of healthy dietary practices among cancer survivors to help promote and encourage HE cross various stages of cancer care on a regular basis. Such training must take into account the most common barriers that these nurses experience regarding their relative lack of HE promotion to cancer survivors.

Among the listed barriers in the survey, there was no clear barrier that most cancer nurses reported as being most important. As a result, barriers reflecting an absence *e.g.*, a lack of expertise, time, knowledge regarding HE habits or support structures for the promotion of HE behaviors, were all relatively equally perceived by the nursing staff. The aforementioned results are consistent with results of previous studies conducted among pediatric nurses as well as Indian nurses, wherein lack of time, knowledge and training regarding HE and PA, respectively were the most commonly barriers (Blake & Patterson, 2015; Pai et al., 2022). Unfortunately, due to this spread of perceived barriers, a multiple number of approaches may need to be incorporated to improve the HE promotion practices of nurses among cancer survivors. Incorporation of nurse education programs may be able to improve their perceived expertise and knowledge regarding HE habits. However, such education programs may have little effect if there continues to be no effective interdisciplinary pathway for the promotion and delivery of healthy behaviors services such as dietary support for the cancer survivors.

While the results of this study add to our understanding of cancer survivorship in India, it is not without its limitations. One of the major limitations may reflect the cross-sectional quantitative survey design used in the study. In particular, such as survey design may have some limitations with respect to the common utilisation of single- over multiple-item analyses as well as lack of rich qualitative data. The other major limitation may be the relatively small sample size when considering the total number of cancer nurses and hospitals in India, which may affect the generalisability of these results to the wider Indian context.

## Conclusion

The results of the current study suggest that Indian nurses believe HE has many benefits for cancer survivors, that they wish to promote this behavior for improved cancer survivorship outcomes, but that they experience many barriers to doing so. The major result of the current study suggests that the Indian nurses consider the dietician/nutritionist followed by the oncologists as the primary health care providers responsible for promoting HE among cancer survivors. Furthermore, there was an even mix of the stage at which the Indian nurses promoted HE, with majority of them promoting HE across all stages of cancer treatment. The most frequently described barriers the nurses felt to the promotion of HE reflected the perceived lack of nutrition knowledge, expertise and support structures. These nurses also believed that additional educational and organizational support was essential in improving the promotion of HE amongst cancer survivors.

The findings of this study may contribute to the existing body of evidence regarding the promotion of HE practices by health care professionals among cancer survivors, particularly in countries such as India where the external validity of the wider cancer survivorship literature is somewhat questionable. It is hoped that the findings of this study might stimulate a range of clinical improvements in the way that HE is promoted and supported in Indian hospitals for cancer survivors. Further research into the practices that Indian nurses use to promote healthy behaviors among cancer survivors, while also highlighting the challenges they face while doing the same, will be required to further improve survivorship outcomes.

This cross-sectional survey used the non-probability–convenience sampling for determination of sample size. As is true for convenience sampling, the estimates derived from convenience samples may be biased (Jager, Putnick & Bornstein, 2017) *i.e.,* the sample estimates of nurses from the two tertiary care hospitals are not completely reflective of the target population of Indian nurses. Hence, future studies could focus on recruiting nurses who are working in hospitals that differ on important sociodemographic characteristics, to gain a better understanding of Indian nurses’ perception regarding healthy eating habits amongst cancer survivors.

##  Supplemental Information

10.7717/peerj.17107/supp-1 Supplemental Information 1 Data sets containing the results of the analysed variables included in the survey Data set consisting of results (Tables 1–5)

10.7717/peerj.17107/supp-2 Supplemental Information 2 Survey Questionaire

10.7717/peerj.17107/supp-3 Supplemental Information 3 Raw data

## Additional Information and Declarations

### Data Availability

The following information was supplied regarding data availability: The results of the data analysis are available in the Supplementary File.
