# Supplementary material for: Beliefs, barriers, and promotion practices of Indian nurses’ regarding healthy eating for cancer survivors in a tertiary care hospital—A cross sectional survey
Source: PeerJ. 2024 Mar 19;12:e17107. doi: 10.7717/peerj.17107 (PMC10959102; doi:10.7717/peerj.17107)
Supplement: Supplemental Information 1 — Data set consisting of results (Tables 1–5) [file peerj-12-17107-s001.docx]

**Descriptives Table 3 and Table 4 HE**

| Descriptives | | | | | | | | | | | | | | | | | | | | | | | | | | | | | |
| --- | --- | --- | --- | --- | --- | --- | --- | --- | --- | --- | --- | --- | --- | --- | --- | --- | --- | --- | --- | --- | --- | --- | --- | --- | --- | --- | --- | --- | --- |
|  |  |  |  |  |  |  |  |  |  |  |  |  |  |  |  |  |  |  |  |  |  |  |  |  |  |  |  |  |  |
|  | | **Q3** | | **17 HRQL** | | **17 WT** | | **17 FATIGUE** | | **17MENTAL** | | **17 ADL** | | **17 CA** | | **17 CHRONIC** | | **17 TUMOR** | | **17 NO B** | | **HEPB 22** | | **24 HE** | | **24 HE (2)** | | **24 EVE HE** | |
| N |  | 1 |  | 178 |  | 175 |  | 178 |  | 174 |  | 177 |  | 174 |  | 175 |  | 173 |  | 161 |  | 171 |  | 174 |  | 173 |  | 173 |  |
|  |  | 2 |  | 201 |  | 203 |  | 201 |  | 198 |  | 200 |  | 198 |  | 200 |  | 200 |  | 176 |  | 201 |  | 197 |  | 203 |  | 200 |  |
| Missing |  | 1 |  | 1 |  | 4 |  | 1 |  | 5 |  | 2 |  | 5 |  | 4 |  | 6 |  | 18 |  | 8 |  | 5 |  | 6 |  | 6 |  |
|  |  | 2 |  | 4 |  | 2 |  | 4 |  | 7 |  | 5 |  | 7 |  | 5 |  | 5 |  | 29 |  | 4 |  | 8 |  | 2 |  | 5 |  |
| Mean |  | 1 |  | 3.00 |  | 3.01 |  | 2.93 |  | 2.95 |  | 2.97 |  | 2.77 |  | 2.85 |  | 2.75 |  | 2.02 |  | 2.44 |  | 2.49 |  | 2.82 |  | 2.74 |  |
|  |  | 2 |  | 2.94 |  | 2.98 |  | 2.95 |  | 3.02 |  | 3.00 |  | 2.96 |  | 2.92 |  | 2.79 |  | 2.14 |  | 2.53 |  | 2.74 |  | 2.87 |  | 2.83 |  |
| Standard deviation |  | 1 |  | 0.729 |  | 0.743 |  | 0.752 |  | 0.691 |  | 0.749 |  | 0.756 |  | 0.781 |  | 0.817 |  | 0.965 |  | 0.783 |  | 0.838 |  | 0.776 |  | 0.847 |  |
|  |  | 2 |  | 0.592 |  | 0.648 |  | 0.638 |  | 0.587 |  | 0.661 |  | 0.620 |  | 0.697 |  | 0.739 |  | 0.949 |  | 0.707 |  | 0.708 |  | 0.655 |  | 0.705 |  |
|  | | | | | | | | | | | | | | | | | | | | | | | | | | | | | |

**Mann-Whitney U test - healthy eating (Table 4)**

| Independent Samples T-Test | | | | | | | |
| --- | --- | --- | --- | --- | --- | --- | --- |
|  |  |  |  |  |  |  |  |
|  | |  | | **Statistic** | | **p** | |
| 17 HRQL |  | Mann-Whitney U |  | 16570 |  | 0.100 |  |
| 17 WT |  | Mann-Whitney U |  | 17055 |  | 0.418 |  |
| 17 FATIGUE |  | Mann-Whitney U |  | 17682 |  | 0.814 |  |
| 17MENTAL |  | Mann-Whitney U |  | 16453 |  | 0.332 |  |
| 17 ADL |  | Mann-Whitney U |  | 17616 |  | 0.921 |  |
| 17 CA |  | Mann-Whitney U |  | 14667 |  | 0.004 |  |
| 17 CHRONIC |  | Mann-Whitney U |  | 16646 |  | 0.347 |  |
| 17 TUMOR |  | Mann-Whitney U |  | 16475 |  | 0.370 |  |
| 17 NO B |  | Mann-Whitney U |  | 13098 |  | 0.207 |  |
| HEPB 22 |  | Mann-Whitney U |  | 16240 |  | 0.310 |  |
| 24 HE |  | Mann-Whitney U |  | 14347 |  | 0.002 |  |
| 24 HE (2) |  | Mann-Whitney U |  | 17219 |  | 0.697 |  |
| 24 EVE HE |  | Mann-Whitney U |  | 16635 |  | 0.449 |  |
|  | | | | | | | |

# Q17  HE frequencies

| Descriptives | | | | | | | | | | | | | | | | | | | |
| --- | --- | --- | --- | --- | --- | --- | --- | --- | --- | --- | --- | --- | --- | --- | --- | --- | --- | --- | --- |
|  |  |  |  |  |  |  |  |  |  |  |  |  |  |  |  |  |  |  |  |
|  | | **17 HRQL** | | **17 WT** | | **17 FATIGUE** | | **17MENTAL** | | **17 ADL** | | **17 CA** | | **17 CHRONIC** | | **17 NO B** | | **17 TUMOR** | |
| N |  | 383 |  | 382 |  | 383 |  | 376 |  | 381 |  | 376 |  | 379 |  | 341 |  | 377 |  |
| Missing |  | 5 |  | 6 |  | 5 |  | 12 |  | 7 |  | 12 |  | 9 |  | 47 |  | 11 |  |
| Mean |  | 2.97 |  | 2.99 |  | 2.94 |  | 2.99 |  | 2.98 |  | 2.87 |  | 2.89 |  | 2.09 |  | 2.77 |  |
| Standard deviation |  | 0.656 |  | 0.689 |  | 0.690 |  | 0.637 |  | 0.699 |  | 0.689 |  | 0.733 |  | 0.954 |  | 0.773 |  |
|  | | | | | | | | | | | | | | | | | | | |

## Frequencies

| Frequencies of 17 HRQL | | | | | | | |
| --- | --- | --- | --- | --- | --- | --- | --- |
|  |  |  |  |  |  |  |  |
| **Levels** | | **Counts** | | **% of Total** | | **Cumulative %** | |
| 1 |  | 24 |  | 6.3 % |  | 6.3 % |  |
| 2 |  | 17 |  | 4.4 % |  | 10.7 % |  |
| 3 |  | 290 |  | 75.7 % |  | 86.4 % |  |
| 4 |  | 52 |  | 13.6 % |  | 100.0 % |  |
|  | | | | | | | |

| Frequencies of 17 WT | | | | | | | |
| --- | --- | --- | --- | --- | --- | --- | --- |
|  |  |  |  |  |  |  |  |
| **Levels** | | **Counts** | | **% of Total** | | **Cumulative %** | |
| 1 |  | 20 |  | 5.2 % |  | 5.2 % |  |
| 2 |  | 32 |  | 8.4 % |  | 13.6 % |  |
| 3 |  | 261 |  | 68.3 % |  | 81.9 % |  |
| 4 |  | 69 |  | 18.1 % |  | 100.0 % |  |
|  | | | | | | | |

| Frequencies of 17 FATIGUE | | | | | | | |
| --- | --- | --- | --- | --- | --- | --- | --- |
|  |  |  |  |  |  |  |  |
| **Levels** | | **Counts** | | **% of Total** | | **Cumulative %** | |
| 1 |  | 20 |  | 5.2 % |  | 5.2 % |  |
| 2 |  | 43 |  | 11.2 % |  | 16.4 % |  |
| 3 |  | 260 |  | 67.9 % |  | 84.3 % |  |
| 4 |  | 60 |  | 15.7 % |  | 100.0 % |  |
|  | | | | | | | |

| Frequencies of 17MENTAL | | | | | | | |
| --- | --- | --- | --- | --- | --- | --- | --- |
|  |  |  |  |  |  |  |  |
| **Levels** | | **Counts** | | **% of Total** | | **Cumulative %** | |
| 1 |  | 18 |  | 4.8 % |  | 4.8 % |  |
| 2 |  | 24 |  | 6.4 % |  | 11.2 % |  |
| 3 |  | 278 |  | 73.9 % |  | 85.1 % |  |
| 4 |  | 56 |  | 14.9 % |  | 100.0 % |  |
|  | | | | | | | |

| Frequencies of 17 ADL | | | | | | | |
| --- | --- | --- | --- | --- | --- | --- | --- |
|  |  |  |  |  |  |  |  |
| **Levels** | | **Counts** | | **% of Total** | | **Cumulative %** | |
| 1 |  | 25 |  | 6.6 % |  | 6.6 % |  |
| 2 |  | 21 |  | 5.5 % |  | 12.1 % |  |
| 3 |  | 270 |  | 70.9 % |  | 82.9 % |  |
| 4 |  | 65 |  | 17.1 % |  | 100.0 % |  |
|  | | | | | | | |

| Frequencies of 17 CA | | | | | | | |
| --- | --- | --- | --- | --- | --- | --- | --- |
|  |  |  |  |  |  |  |  |
| **Levels** | | **Counts** | | **% of Total** | | **Cumulative %** | |
| 1 |  | 17 |  | 4.5 % |  | 4.5 % |  |
| 2 |  | 65 |  | 17.3 % |  | 21.8 % |  |
| 3 |  | 243 |  | 64.6 % |  | 86.4 % |  |
| 4 |  | 51 |  | 13.6 % |  | 100.0 % |  |
|  | | | | | | | |

| Frequencies of 17 CHRONIC | | | | | | | |
| --- | --- | --- | --- | --- | --- | --- | --- |
|  |  |  |  |  |  |  |  |
| **Levels** | | **Counts** | | **% of Total** | | **Cumulative %** | |
| 1 |  | 22 |  | 5.8 % |  | 5.8 % |  |
| 2 |  | 59 |  | 15.6 % |  | 21.4 % |  |
| 3 |  | 237 |  | 62.5 % |  | 83.9 % |  |
| 4 |  | 61 |  | 16.1 % |  | 100.0 % |  |
|  | | | | | | | |

| Frequencies of 17 NO B | | | | | | | |
| --- | --- | --- | --- | --- | --- | --- | --- |
|  |  |  |  |  |  |  |  |
| **Levels** | | **Counts** | | **% of Total** | | **Cumulative %** | |
| 1 |  | 122 |  | 35.8 % |  | 35.8 % |  |
| 2 |  | 86 |  | 25.2 % |  | 61.0 % |  |
| 3 |  | 114 |  | 33.4 % |  | 94.4 % |  |
| 4 |  | 19 |  | 5.6 % |  | 100.0 % |  |
|  | | | | | | | |

| Frequencies of 17 TUMOR | | | | | | | |
| --- | --- | --- | --- | --- | --- | --- | --- |
|  |  |  |  |  |  |  |  |
| **Levels** | | **Counts** | | **% of Total** | | **Cumulative %** | |
| 1 |  | 30 |  | 8.0 % |  | 8.0 % |  |
| 2 |  | 75 |  | 19.9 % |  | 27.9 % |  |
| 3 |  | 223 |  | 59.2 % |  | 87.0 % |  |
| 4 |  | 49 |  | 13.0 % |  | 100.0 % |  |
|  | | | | | | | |

# Q22  HE frequencies

| Descriptives | | | |
| --- | --- | --- | --- |
|  |  |  |  |
|  | | **HEPB 22** | |
| N |  | 376 |  |
| Missing |  | 12 |  |
|  | | | |

## Frequencies

| Frequencies of HEPB 22 | | | | | | | |
| --- | --- | --- | --- | --- | --- | --- | --- |
|  |  |  |  |  |  |  |  |
| **Levels** | | **Counts** | | **% of Total** | | **Cumulative %** | |
| 1 |  | 45 |  | 12.0 % |  | 12.0 % |  |
| 2 |  | 115 |  | 30.6 % |  | 42.6 % |  |
| 3 |  | 204 |  | 54.3 % |  | 96.8 % |  |
| 4 |  | 12 |  | 3.2 % |  | 100.0 % |  |
|  | | | | | | | |

# Frequencies Q24 HE

| Descriptives | | | | | | | |
| --- | --- | --- | --- | --- | --- | --- | --- |
|  |  |  |  |  |  |  |  |
|  | | **24 HE** | | **24 HE (2)** | | **24 EVE HE** | |
| N |  | 374 |  | 379 |  | 376 |  |
| Missing |  | 14 |  | 9 |  | 12 |  |
|  | | | | | | | |

## Frequencies

| Frequencies of 24 HE | | | | | | | |
| --- | --- | --- | --- | --- | --- | --- | --- |
|  |  |  |  |  |  |  |  |
| **Levels** | | **Counts** | | **% of Total** | | **Cumulative %** | |
| 1 |  | 42 |  | 11.2 % |  | 11.2 % |  |
| 2 |  | 85 |  | 22.7 % |  | 34.0 % |  |
| 3 |  | 220 |  | 58.8 % |  | 92.8 % |  |
| 4 |  | 27 |  | 7.2 % |  | 100.0 % |  |
|  | | | | | | | |

| Frequencies of 24 HE (2) | | | | | | | |
| --- | --- | --- | --- | --- | --- | --- | --- |
|  |  |  |  |  |  |  |  |
| **Levels** | | **Counts** | | **% of Total** | | **Cumulative %** | |
| 1 |  | 25 |  | 6.6 % |  | 6.6 % |  |
| 2 |  | 54 |  | 14.2 % |  | 20.8 % |  |
| 3 |  | 254 |  | 67.0 % |  | 87.9 % |  |
| 4 |  | 46 |  | 12.1 % |  | 100.0 % |  |
|  | | | | | | | |

| Frequencies of 24 EVE HE | | | | | | | |
| --- | --- | --- | --- | --- | --- | --- | --- |
|  |  |  |  |  |  |  |  |
| **Levels** | | **Counts** | | **% of Total** | | **Cumulative %** | |
| 1 |  | 37 |  | 9.8 % |  | 9.8 % |  |
| 2 |  | 49 |  | 13.0 % |  | 22.9 % |  |
| 3 |  | 247 |  | 65.7 % |  | 88.6 % |  |
| 4 |  | 43 |  | 11.4 % |  | 100.0 % |  |
|  | | | | | | | |

Demographic characteristics of the nurses

# Descriptives – demographics Table 1

Q 1,2,3,4,5,6,7

| Descriptives | | | | | | | | | | | | | | | |
| --- | --- | --- | --- | --- | --- | --- | --- | --- | --- | --- | --- | --- | --- | --- | --- |
|  |  |  |  |  |  |  |  |  |  |  |  |  |  |  |  |
|  | | **AGE-ANALYSIS** | | **GENDER** | | **Q3** | | **YOP** | | **Q5 Cat** | | **Q6** | | **LOCATION** | |
| N |  | 384 |  | 388 |  | 384 |  | 377 |  | 388 |  | 380 |  | 382 |  |
| Missing |  | 4 |  | 0 |  | 4 |  | 11 |  | 0 |  | 8 |  | 6 |  |
| Shapiro-Wilk W |  | . |  | . |  | . |  | . |  | . |  | . |  | . |  |
| Shapiro-Wilk p |  |  |  |  |  |  |  |  |  |  |  |  |  |  |  |
|  | | | | | | | | | | | | | | | |

## Frequencies

| Frequencies of AGE-ANALYSIS | | | | | | | |
| --- | --- | --- | --- | --- | --- | --- | --- |
|  |  |  |  |  |  |  |  |
| **Levels** | | **Counts** | | **% of Total** | | **Cumulative %** | |
| LESSTHAN25 |  | 80 |  | 20.8 % |  | 20.8 % |  |
| LESSTHAN35 |  | 166 |  | 43.2 % |  | 64.1 % |  |
| LESSTHAN45 |  | 76 |  | 19.8 % |  | 83.9 % |  |
| LESSTHAN55 |  | 55 |  | 14.3 % |  | 98.2 % |  |
| LESSTHAN65 |  | 7 |  | 1.8 % |  | 100.0 % |  |
|  | | | | | | | |

| Frequencies of GENDER | | | | | | | |
| --- | --- | --- | --- | --- | --- | --- | --- |
|  |  |  |  |  |  |  |  |
| **Levels** | | **Counts** | | **% of Total** | | **Cumulative %** | |
| F |  | 379 |  | 97.7 % |  | 97.7 % |  |
| M |  | 9 |  | 2.3 % |  | 100.0 % |  |
|  | | | | | | | |

| Frequencies of Q3 | | | | | | | |
| --- | --- | --- | --- | --- | --- | --- | --- |
|  |  |  |  |  |  |  |  |
| **Levels** | | **Counts** | | **% of Total** | | **Cumulative %** | |
| BSC NURSING |  | 179 |  | 46.6 % |  | 46.6 % |  |
| GNM |  | 205 |  | 53.4 % |  | 100.0 % |  |
|  | | | | | | | |

| Frequencies of YOP | | | | | | | |
| --- | --- | --- | --- | --- | --- | --- | --- |
|  |  |  |  |  |  |  |  |
| **Levels** | | **Counts** | | **% of Total** | | **Cumulative %** | |
| LESSTHAN5 |  | 102 |  | 27.1 % |  | 27.1 % |  |
| LESSTHAN14 |  | 170 |  | 45.1 % |  | 72.1 % |  |
| LESSTHAN24 |  | 53 |  | 14.1 % |  | 86.2 % |  |
| MORETHAN25 |  | 52 |  | 13.8 % |  | 100.0 % |  |
|  | | | | | | | |

| Frequencies of Q5 Cat | | | | | | | |
| --- | --- | --- | --- | --- | --- | --- | --- |
|  |  |  |  |  |  |  |  |
| **Levels** | | **Counts** | | **% of Total** | | **Cumulative %** | |
| 1 |  | 21 |  | 5.4 % |  | 5.4 % |  |
| 10 |  | 17 |  | 4.4 % |  | 9.8 % |  |
| 11 |  | 1 |  | 0.3 % |  | 10.1 % |  |
| 12 |  | 1 |  | 0.3 % |  | 10.3 % |  |
| 13 |  | 1 |  | 0.3 % |  | 10.6 % |  |
| 14 |  | 1 |  | 0.3 % |  | 10.8 % |  |
| 15 |  | 3 |  | 0.8 % |  | 11.6 % |  |
| 16 |  | 1 |  | 0.3 % |  | 11.9 % |  |
| 2 |  | 19 |  | 4.9 % |  | 16.8 % |  |
| 20 |  | 3 |  | 0.8 % |  | 17.5 % |  |
| 25 |  | 2 |  | 0.5 % |  | 18.0 % |  |
| 3 |  | 18 |  | 4.6 % |  | 22.7 % |  |
| 4 |  | 13 |  | 3.4 % |  | 26.0 % |  |
| 5 |  | 17 |  | 4.4 % |  | 30.4 % |  |
| 6 |  | 12 |  | 3.1 % |  | 33.5 % |  |
| 7 |  | 2 |  | 0.5 % |  | 34.0 % |  |
| 8 |  | 4 |  | 1.0 % |  | 35.1 % |  |
| NIL |  | 252 |  | 64.9 % |  | 100.0 % |  |
|  | | | | | | | |

| Frequencies of Q6 | | | | | | | |
| --- | --- | --- | --- | --- | --- | --- | --- |
|  |  |  |  |  |  |  |  |
| **Levels** | | **Counts** | | **% of Total** | | **Cumulative %** | |
| PRIVATE |  | 375 |  | 98.7 % |  | 98.7 % |  |
| PUBLIC |  | 5 |  | 1.3 % |  | 100.0 % |  |
|  | | | | | | | |

| Frequencies of LOCATION | | | | | | | |
| --- | --- | --- | --- | --- | --- | --- | --- |
|  |  |  |  |  |  |  |  |
| **Levels** | | **Counts** | | **% of Total** | | **Cumulative %** | |
| METRO |  | 55 |  | 14.4 % |  | 14.4 % |  |
| REGIONAL |  | 283 |  | 74.1 % |  | 88.5 % |  |
| RURAL |  | 44 |  | 11.5 % |  | 100.0 % |  |
|  | | | | | | | |

# Descriptives -  Healthy eating

EAT HEALTHY REGULARLY - Q 13

| Descriptives | | | |
| --- | --- | --- | --- |
|  |  |  |  |
|  | | **EAT HEALTHY** | |
| N |  | 382 |  |
| Missing |  | 6 |  |
|  | | | |

## Frequencies

| Frequencies of EAT HEALTHY | | | | | | | |
| --- | --- | --- | --- | --- | --- | --- | --- |
|  |  |  |  |  |  |  |  |
| **Levels** | | **Counts** | | **% of Total** | | **Cumulative %** | |
| YES |  | 322 |  | 84.3 % |  | 84.3 % |  |
| NO |  | 60 |  | 15.7 % |  | 100.0 % |  |
|  | | | | | | | |

# Descriptive – Table 2

Q10-PRIMARY PERSON RESPONSIBLE FOR PROMOTING HEALTHY EATING &
Q11-STAGE AT WHICH HEALTHY EATING IS PROMOTED

| Descriptives | | | |
| --- | --- | --- | --- |
|  |  |  |  |
|  | | **PHR10-PRIMARY PERSON RESPONSIBLE FOR PROMOTING HEALTHY EATING** | |
| N |  | 386 |  |
| Missing |  | 2 |  |
|  | | | |

## Frequencies

| Frequencies of PHR10-PRIMARY PERSON RESPONSIBLE FOR PROMOTING HEALTHY EATING | | | | | | | |
| --- | --- | --- | --- | --- | --- | --- | --- |
|  |  |  |  |  |  |  |  |
| **Levels** | | **Counts** | | **% of Total** | | **Cumulative %** | |
| NURSE |  | 12 |  | 3.1 % |  | 3.1 % |  |
| PT |  | 21 |  | 5.4 % |  | 8.5 % |  |
| ONCOLOGIST |  | 40 |  | 10.4 % |  | 18.9 % |  |
| EXE PHY |  | 3 |  | 0.8 % |  | 19.7 % |  |
| NUTRITIONIST |  | 306 |  | 79.3 % |  | 99.0 % |  |
| DONT KNOW |  | 3 |  | 0.8 % |  | 99.7 % |  |
| OTHERS |  | 1 |  | 0.3 % |  | 100.0 % |  |
|  | | | | | | | |

# Descriptives - Table 2

| Descriptives | | | | | |
| --- | --- | --- | --- | --- | --- |
|  |  |  |  |  |  |
|  | | **Q3** | | **PHR 11** | |
| N |  | BSC NURSING |  | 177 |  |
|  |  | GNM |  | 204 |  |
| Missing |  | BSC NURSING |  | 2 |  |
|  |  | GNM |  | 1 |  |
| Mean |  | BSC NURSING |  |  |  |
|  |  | GNM |  |  |  |
| Median |  | BSC NURSING |  |  |  |
|  |  | GNM |  |  |  |
| Standard deviation |  | BSC NURSING |  |  |  |
|  |  | GNM |  |  |  |
| Minimum |  | BSC NURSING |  |  |  |
|  |  | GNM |  |  |  |
| Maximum |  | BSC NURSING |  |  |  |
|  |  | GNM |  |  |  |
|  | | | | | |

## Frequencies

| Frequencies of PHR 11 | | | | | |
| --- | --- | --- | --- | --- | --- |
|  | | **Q3** | | | |
| **PHR 11** | | **BSC NURSING** | | **GNM** | |
| Pre Rx |  | 40 |  | 45 |  |
| Pre/Post Rx |  | 15 |  | 7 |  |
| Every Stage |  | 32 |  | 33 |  |
| Pre/During Rx |  | 1 |  | 1 |  |
| Post Rx |  | 36 |  | 52 |  |
| During/post Rx |  | 8 |  | 8 |  |
| During Rx |  | 41 |  | 52 |  |
| Dont know |  | 4 |  | 6 |  |
|  | | | | | |

# Contingency Tables - primary person responsible for promoting HE (Table 2)

| Contingency Tables | | | | | | | |
| --- | --- | --- | --- | --- | --- | --- | --- |
|  | | **Q3** | | | |  | |
| **PHR10** | | **BSC NURSING** | | **GNM** | | **Total** | |
| 1 |  | 6 |  | 6 |  | 12 |  |
| 2 |  | 5 |  | 16 |  | 21 |  |
| 3 |  | 23 |  | 15 |  | 38 |  |
| 4 |  | 1 |  | 2 |  | 3 |  |
| 5 |  | 141 |  | 163 |  | 304 |  |
| 6 |  | 1 |  | 2 |  | 3 |  |
| 7 |  | 1 |  | 0 |  | 1 |  |
| Total |  | 178 |  | 204 |  | 382 |  |
|  | | | | | | | |

| χ² Tests | | | | | | | |
| --- | --- | --- | --- | --- | --- | --- | --- |
|  |  |  |  |  |  |  |  |
|  | | **Value** | | **df** | | **p** | |
| χ² |  | 8.98 |  | 6 |  | 0.175 |  |
| N |  | 382 |  |  | |  | |
|  | | | | | | | |

# Table 2

CHI SQUARE-INDICATE THE SATGE AT WHICH HEALTHY EATING IS PROMOTED (Q11)

| Contingency Tables | | | | | | | |
| --- | --- | --- | --- | --- | --- | --- | --- |
|  | | **Q3** | | | |  | |
| **PHR 11** | | **BSC NURSING** | | **GNM** | | **Total** | |
| Pre Rx |  | 40 |  | 45 |  | 85 |  |
| Pre/Post Rx |  | 15 |  | 7 |  | 22 |  |
| Every Stage |  | 32 |  | 33 |  | 65 |  |
| Pre/During Rx |  | 1 |  | 1 |  | 2 |  |
| Post Rx |  | 36 |  | 52 |  | 88 |  |
| During/post Rx |  | 8 |  | 8 |  | 16 |  |
| During Rx |  | 41 |  | 52 |  | 93 |  |
| Dont know |  | 4 |  | 6 |  | 10 |  |
| Total |  | 177 |  | 204 |  | 381 |  |
|  | | | | | | | |

| χ² Tests | | | | | | | |
| --- | --- | --- | --- | --- | --- | --- | --- |
|  |  |  |  |  |  |  |  |
|  | | **Value** | | **df** | | **p** | |
| χ² |  | 5.95 |  | 7 |  | 0.546 |  |
| N |  | 381 |  |  | |  | |
|  | | | | | | | |

**TABLE 5**

# Descriptives - barriers in HEALTHY EATING promotion  (Q21)

| Descriptives | | | | | | | | | | | | | | | | | | | |
| --- | --- | --- | --- | --- | --- | --- | --- | --- | --- | --- | --- | --- | --- | --- | --- | --- | --- | --- | --- |
|  |  |  |  |  |  |  |  |  |  |  |  |  |  |  |  |  |  |  |  |
|  | | **MOT 21 TIME** | | **MOT 21 RISK TO PATEINT** | | **MOT 21 ADEQUATE SS** | | **MOT 21 KNOWLEDGE** | | **MOT 21 EXPERTISE** | | **MOT 21 PROMOTE** | | **MOT 21 JOB** | | **MOT 21 BARRIERS** | | **OTHERS (2)** | |
| N |  | 356 |  | 338 |  | 360 |  | 339 |  | 330 |  | 299 |  | 291 |  | 289 |  | 205 |  |
| Missing |  | 32 |  | 50 |  | 28 |  | 49 |  | 58 |  | 89 |  | 97 |  | 99 |  | 183 |  |
| Mean |  | 1.72 |  | 1.80 |  | 1.78 |  | 1.80 |  | 1.79 |  | 1.91 |  | 2.03 |  |  |  | 1.83 |  |
| Median |  | 2.00 |  | 2.00 |  | 2.00 |  | 2 |  | 2.00 |  | 2 |  | 2 |  |  |  | 2 |  |
| Standard deviation |  | 0.657 |  | 0.674 |  | 0.664 |  | 0.690 |  | 0.678 |  | 0.720 |  | 0.728 |  |  |  | 0.666 |  |
| Shapiro-Wilk W |  | 0.777 |  | 0.791 |  | 0.786 |  | 0.793 |  | 0.790 |  | 0.806 |  | 0.809 |  | . |  | 0.791 |  |
| Shapiro-Wilk p |  | < .001 |  | < .001 |  | < .001 |  | < .001 |  | < .001 |  | < .001 |  | < .001 |  |  |  | < .001 |  |

## Frequencies

| Frequencies of MOT 21 TIME | | | | | | | |
| --- | --- | --- | --- | --- | --- | --- | --- |
|  |  |  |  |  |  |  |  |
| **Levels** | | **Counts** | | **% of Total** | | **Cumulative %** | |
| most likely |  | 139 |  | 39.0 % |  | 39.0 % |  |
| neutral |  | 176 |  | 49.4 % |  | 88.5 % |  |
| least likely |  | 41 |  | 11.5 % |  | 100.0 % |  |
|  | | | | | | | |

| Frequencies of MOT 21 RISK TO PATEINT | | | | | | | |
| --- | --- | --- | --- | --- | --- | --- | --- |
|  |  |  |  |  |  |  |  |
| **Levels** | | **Counts** | | **% of Total** | | **Cumulative %** | |
| most likely |  | 116 |  | 34.3 % |  | 34.3 % |  |
| neutral |  | 172 |  | 50.9 % |  | 85.2 % |  |
| least likely |  | 50 |  | 14.8 % |  | 100.0 % |  |
|  | | | | | | | |

| Frequencies of MOT 21 ADEQUATE SS | | | | | | | |
| --- | --- | --- | --- | --- | --- | --- | --- |
|  |  |  |  |  |  |  |  |
| **Levels** | | **Counts** | | **% of Total** | | **Cumulative %** | |
| most likely |  | 128 |  | 35.6 % |  | 35.6 % |  |
| neutral |  | 184 |  | 51.1 % |  | 86.7 % |  |
| least likely |  | 48 |  | 13.3 % |  | 100.0 % |  |
|  | | | | | | | |

| Frequencies of MOT 21 KNOWLEDGE | | | | | | | |
| --- | --- | --- | --- | --- | --- | --- | --- |
|  |  |  |  |  |  |  |  |
| **Levels** | | **Counts** | | **% of Total** | | **Cumulative %** | |
| most likely |  | 122 |  | 36.0 % |  | 36.0 % |  |
| neutral |  | 164 |  | 48.4 % |  | 84.4 % |  |
| least likely |  | 53 |  | 15.6 % |  | 100.0 % |  |
|  | | | | | | | |

| Frequencies of MOT 21 EXPERTISE | | | | | | | |
| --- | --- | --- | --- | --- | --- | --- | --- |
|  |  |  |  |  |  |  |  |
| **Levels** | | **Counts** | | **% of Total** | | **Cumulative %** | |
| most likely |  | 118 |  | 35.8 % |  | 35.8 % |  |
| neutral |  | 164 |  | 49.7 % |  | 85.5 % |  |
| least likely |  | 48 |  | 14.5 % |  | 100.0 % |  |
|  | | | | | | | |

| Frequencies of MOT 21 PROMOTE | | | | | | | |
| --- | --- | --- | --- | --- | --- | --- | --- |
|  |  |  |  |  |  |  |  |
| **Levels** | | **Counts** | | **% of Total** | | **Cumulative %** | |
| most likely |  | 92 |  | 30.8 % |  | 30.8 % |  |
| neutral |  | 142 |  | 47.5 % |  | 78.3 % |  |
| least likely |  | 65 |  | 21.7 % |  | 100.0 % |  |
|  | | | | | | | |

| Frequencies of MOT 21 JOB | | | | | | | |
| --- | --- | --- | --- | --- | --- | --- | --- |
|  |  |  |  |  |  |  |  |
| **Levels** | | **Counts** | | **% of Total** | | **Cumulative %** | |
| most likely |  | 73 |  | 25.1 % |  | 25.1 % |  |
| neutral |  | 137 |  | 47.1 % |  | 72.2 % |  |
| least likely |  | 81 |  | 27.8 % |  | 100.0 % |  |
|  | | | | | | | |

| Frequencies of MOT 21 BARRIERS | | | | | | | |
| --- | --- | --- | --- | --- | --- | --- | --- |
|  |  |  |  |  |  |  |  |
| **Levels** | | **Counts** | | **% of Total** | | **Cumulative %** | |
| most likely |  | 79 |  | 27.3 % |  | 27.3 % |  |
| neutral |  | 148 |  | 51.2 % |  | 78.5 % |  |
| least likely |  | 62 |  | 21.5 % |  | 100.0 % |  |
|  | | | | | | | |

| Frequencies of OTHERS (2) | | | | | | | |
| --- | --- | --- | --- | --- | --- | --- | --- |
|  |  |  |  |  |  |  |  |
| **Levels** | | **Counts** | | **% of Total** | | **Cumulative %** | |
| most likely |  | 65 |  | 31.7 % |  | 31.7 % |  |
| neutral |  | 109 |  | 53.2 % |  | 84.9 % |  |
| least likely |  | 31 |  | 15.1 % |  | 100.0 % |  |
|  | | | | | | | |

# Chi square (Q21) BARRIERS IN PROMOTING HEALTHY EATING

CHI SQUARE - LACK OF TIME

| Contingency Tables | | | | | | | | | |
| --- | --- | --- | --- | --- | --- | --- | --- | --- | --- |
|  | | | | **Q3** | | | |  | |
| **MOT 21 TIME** | |  | | **BSC NURSING** | | **GNM** | | **Total** | |
| most likely |  | Observed |  | 61 |  | 78 |  | 139 |  |
|  | | % of total |  | 17.3 % |  | 22.2 % |  | 39.5 % |  |
| neutral |  | Observed |  | 81 |  | 91 |  | 172 |  |
|  | | % of total |  | 23.0 % |  | 25.9 % |  | 48.9 % |  |
| least likely |  | Observed |  | 21 |  | 20 |  | 41 |  |
|  | | % of total |  | 6.0 % |  | 5.7 % |  | 11.6 % |  |
| Total |  | Observed |  | 163 |  | 189 |  | 352 |  |
|  | | % of total |  | 46.3 % |  | 53.7 % |  | 100.0 % |  |
|  | | | | | | | | | |

| χ² Tests | | | | | | | |
| --- | --- | --- | --- | --- | --- | --- | --- |
|  |  |  |  |  |  |  |  |
|  | | **Value** | | **df** | | **p** | |
| χ² |  | 0.769 |  | 2 |  | 0.681 |  |
| N |  | 352 |  |  | |  | |
|  | | | | | | | |

# Chi Square - risk to patient (HE)

| Contingency Tables | | | | | | | | | |
| --- | --- | --- | --- | --- | --- | --- | --- | --- | --- |
|  | | | | **Q3** | | | |  | |
| **MOT 21 RISK TO PATEINT** | |  | | **BSC NURSING** | | **GNM** | | **Total** | |
| most likely |  | Observed |  | 56 |  | 60 |  | 116 |  |
|  | | % of total |  | 16.8 % |  | 18.0 % |  | 34.7 % |  |
| neutral |  | Observed |  | 73 |  | 95 |  | 168 |  |
|  | | % of total |  | 21.9 % |  | 28.4 % |  | 50.3 % |  |
| least likely |  | Observed |  | 22 |  | 28 |  | 50 |  |
|  | | % of total |  | 6.6 % |  | 8.4 % |  | 15.0 % |  |
| Total |  | Observed |  | 151 |  | 183 |  | 334 |  |
|  | | % of total |  | 45.2 % |  | 54.8 % |  | 100.0 % |  |
|  | | | | | | | | | |

| χ² Tests | | | | | | | |
| --- | --- | --- | --- | --- | --- | --- | --- |
|  |  |  |  |  |  |  |  |
|  | | **Value** | | **df** | | **p** | |
| χ² |  | 0.679 |  | 2 |  | 0.712 |  |
| N |  | 334 |  |  | |  | |
|  | | | | | | | |

# Chi Square - lack of adeqaute support structure

| Contingency Tables | | | | | | | | | |
| --- | --- | --- | --- | --- | --- | --- | --- | --- | --- |
|  | | | | **Q3** | | | |  | |
| **MOT 21 ADEQUATE SS** | |  | | **BSC NURSING** | | **GNM** | | **Total** | |
| most likely |  | Observed |  | 57 |  | 71 |  | 128 |  |
|  | | % of total |  | 16.0 % |  | 19.9 % |  | 36.0 % |  |
| neutral |  | Observed |  | 91 |  | 89 |  | 180 |  |
|  | | % of total |  | 25.6 % |  | 25.0 % |  | 50.6 % |  |
| least likely |  | Observed |  | 21 |  | 27 |  | 48 |  |
|  | | % of total |  | 5.9 % |  | 7.6 % |  | 13.5 % |  |
| Total |  | Observed |  | 169 |  | 187 |  | 356 |  |
|  | | % of total |  | 47.5 % |  | 52.5 % |  | 100.0 % |  |
|  | | | | | | | | | |

| χ² Tests | | | | | | | |
| --- | --- | --- | --- | --- | --- | --- | --- |
|  |  |  |  |  |  |  |  |
|  | | **Value** | | **df** | | **p** | |
| χ² |  | 1.40 |  | 2 |  | 0.497 |  |
| N |  | 356 |  |  | |  | |
|  | | | | | | | |

# Chi Square - lack of knowledge (HE)

| Contingency Tables | | | | | | | | | |
| --- | --- | --- | --- | --- | --- | --- | --- | --- | --- |
|  | | | | **Q3** | | | |  | |
| **MOT 21 KNOWLEDGE** | |  | | **BSC NURSING** | | **GNM** | | **Total** | |
| most likely |  | Observed |  | 49 |  | 73 |  | 122 |  |
|  | | % of total |  | 14.6 % |  | 21.8 % |  | 36.4 % |  |
| neutral |  | Observed |  | 74 |  | 86 |  | 160 |  |
|  | | % of total |  | 22.1 % |  | 25.7 % |  | 47.8 % |  |
| least likely |  | Observed |  | 26 |  | 27 |  | 53 |  |
|  | | % of total |  | 7.8 % |  | 8.1 % |  | 15.8 % |  |
| Total |  | Observed |  | 149 |  | 186 |  | 335 |  |
|  | | % of total |  | 44.5 % |  | 55.5 % |  | 100.0 % |  |
|  | | | | | | | | | |

| χ² Tests | | | | | | | |
| --- | --- | --- | --- | --- | --- | --- | --- |
|  |  |  |  |  |  |  |  |
|  | | **Value** | | **df** | | **p** | |
| χ² |  | 1.57 |  | 2 |  | 0.455 |  |
| N |  | 335 |  |  | |  | |
|  | | | | | | | |

# Chi Square - lack of expertise (HE)

| Contingency Tables | | | | | | | | | |
| --- | --- | --- | --- | --- | --- | --- | --- | --- | --- |
|  | | | | **Q3** | | | |  | |
| **MOT 21 EXPERTISE** | |  | | **BSC NURSING** | | **GNM** | | **Total** | |
| most likely |  | Observed |  | 59 |  | 59 |  | 118 |  |
|  | | % of total |  | 18.1 % |  | 18.1 % |  | 36.2 % |  |
| neutral |  | Observed |  | 72 |  | 88 |  | 160 |  |
|  | | % of total |  | 22.1 % |  | 27.0 % |  | 49.1 % |  |
| least likely |  | Observed |  | 24 |  | 24 |  | 48 |  |
|  | | % of total |  | 7.4 % |  | 7.4 % |  | 14.7 % |  |
| Total |  | Observed |  | 155 |  | 171 |  | 326 |  |
|  | | % of total |  | 47.5 % |  | 52.5 % |  | 100.0 % |  |
|  | | | | | | | | | |

| χ² Tests | | | | | | | |
| --- | --- | --- | --- | --- | --- | --- | --- |
|  |  |  |  |  |  |  |  |
|  | | **Value** | | **df** | | **p** | |
| χ² |  | 0.817 |  | 2 |  | 0.665 |  |
| N |  | 326 |  |  | |  | |
|  | | | | | | | |

# Chi square- i do not promote (HE)

| Contingency Tables | | | | | | | | | |
| --- | --- | --- | --- | --- | --- | --- | --- | --- | --- |
|  | | | | **Q3** | | | |  | |
| **MOT 21 PROMOTE** | |  | | **BSC NURSING** | | **GNM** | | **Total** | |
| most likely |  | Observed |  | 36 |  | 56 |  | 92 |  |
|  | | % of total |  | 12.2 % |  | 19.0 % |  | 31.2 % |  |
| neutral |  | Observed |  | 59 |  | 80 |  | 139 |  |
|  | | % of total |  | 20.0 % |  | 27.1 % |  | 47.1 % |  |
| least likely |  | Observed |  | 35 |  | 29 |  | 64 |  |
|  | | % of total |  | 11.9 % |  | 9.8 % |  | 21.7 % |  |
| Total |  | Observed |  | 130 |  | 165 |  | 295 |  |
|  | | % of total |  | 44.1 % |  | 55.9 % |  | 100.0 % |  |
|  | | | | | | | | | |

| χ² Tests | | | | | | | |
| --- | --- | --- | --- | --- | --- | --- | --- |
|  |  |  |  |  |  |  |  |
|  | | **Value** | | **df** | | **p** | |
| χ² |  | 3.99 |  | 2 |  | 0.136 |  |
| N |  | 295 |  |  | |  | |
|  | | | | | | | |

# Chi square - not my job (HE)

| Contingency Tables | | | | | | | | | |
| --- | --- | --- | --- | --- | --- | --- | --- | --- | --- |
|  | | | | **Q3** | | | |  | |
| **MOT 21 JOB** | |  | | **BSC NURSING** | | **GNM** | | **Total** | |
| most likely |  | Observed |  | 31 |  | 42 |  | 73 |  |
|  | | % of total |  | 10.8 % |  | 14.6 % |  | 25.4 % |  |
| neutral |  | Observed |  | 59 |  | 74 |  | 133 |  |
|  | | % of total |  | 20.6 % |  | 25.8 % |  | 46.3 % |  |
| least likely |  | Observed |  | 42 |  | 39 |  | 81 |  |
|  | | % of total |  | 14.6 % |  | 13.6 % |  | 28.2 % |  |
| Total |  | Observed |  | 132 |  | 155 |  | 287 |  |
|  | | % of total |  | 46.0 % |  | 54.0 % |  | 100.0 % |  |
|  | | | | | | | | | |

| χ² Tests | | | | | | | |
| --- | --- | --- | --- | --- | --- | --- | --- |
|  |  |  |  |  |  |  |  |
|  | | **Value** | | **df** | | **p** | |
| χ² |  | 1.63 |  | 2 |  | 0.443 |  |
| N |  | 287 |  |  | |  | |
|  | | | | | | | |

# Chi Square - i do not have barriers in promoting HE

| Contingency Tables | | | | | | | | | |
| --- | --- | --- | --- | --- | --- | --- | --- | --- | --- |
|  | | | | **Q3** | | | |  | |
| **MOT 21 BARRIERS** | |  | | **BSC NURSING** | | **GNM** | | **Total** | |
| most likely |  | Observed |  | 34 |  | 45 |  | 79 |  |
|  | | % of total |  | 11.9 % |  | 15.8 % |  | 27.7 % |  |
| neutral |  | Observed |  | 65 |  | 79 |  | 144 |  |
|  | | % of total |  | 22.8 % |  | 27.7 % |  | 50.5 % |  |
| least likely |  | Observed |  | 29 |  | 33 |  | 62 |  |
|  | | % of total |  | 10.2 % |  | 11.6 % |  | 21.8 % |  |
| Total |  | Observed |  | 128 |  | 157 |  | 285 |  |
|  | | % of total |  | 44.9 % |  | 55.1 % |  | 100.0 % |  |
|  | | | | | | | | | |

| χ² Tests | | | | | | | |
| --- | --- | --- | --- | --- | --- | --- | --- |
|  |  |  |  |  |  |  |  |
|  | | **Value** | | **df** | | **p** | |
| χ² |  | 0.202 |  | 2 |  | 0.904 |  |
| N |  | 285 |  |  | |  | |
|  | | | | | | | |

# Chi Square - others (HE)

| Contingency Tables | | | | | | | | | |
| --- | --- | --- | --- | --- | --- | --- | --- | --- | --- |
|  | | | | **Q3** | | | |  | |
| **OTHERS (2)** | |  | | **BSC NURSING** | | **GNM** | | **Total** | |
| most likely |  | Observed |  | 29 |  | 36 |  | 65 |  |
|  | | % of total |  | 14.4 % |  | 17.9 % |  | 32.3 % |  |
| neutral |  | Observed |  | 48 |  | 57 |  | 105 |  |
|  | | % of total |  | 23.9 % |  | 28.4 % |  | 52.2 % |  |
| least likely |  | Observed |  | 15 |  | 16 |  | 31 |  |
|  | | % of total |  | 7.5 % |  | 8.0 % |  | 15.4 % |  |
| Total |  | Observed |  | 92 |  | 109 |  | 201 |  |
|  | | % of total |  | 45.8 % |  | 54.2 % |  | 100.0 % |  |
|  | | | | | | | | | |

| χ² Tests | | | | | | | |
| --- | --- | --- | --- | --- | --- | --- | --- |
|  |  |  |  |  |  |  |  |
|  | | **Value** | | **df** | | **p** | |
| χ² |  | 0.121 |  | 2 |  | 0.941 |  |
| N |  | 201 |  |  | |  | |
|  | | | | | | | |
| Table 4 HE  \| Descriptives \| \| \| \| \| \| \| \| \| \| \| \| \| \| \| \| \| \| \| \| \| --- \| --- \| --- \| --- \| --- \| --- \| --- \| --- \| --- \| --- \| --- \| --- \| --- \| --- \| --- \| --- \| --- \| --- \| --- \| --- \| \|  \| \| **17 HRQL** \| \| **17 WT** \| \| **17 FATIGUE** \| \| **17MENTAL** \| \| **17 ADL** \| \| **17 CA** \| \| **17 CHRONIC** \| \| **17 TUMOR** \| \| **17 NO B** \| \| \| N \|  \| 383 \|  \| 382 \|  \| 383 \|  \| 376 \|  \| 381 \|  \| 376 \|  \| 379 \|  \| 377 \|  \| 341 \|  \| \| Missing \|  \| 5 \|  \| 6 \|  \| 5 \|  \| 12 \|  \| 7 \|  \| 12 \|  \| 9 \|  \| 11 \|  \| 47 \|  \| \| Mean \|  \| 2.97 \|  \| 2.99 \|  \| 2.94 \|  \| 2.99 \|  \| 2.98 \|  \| 2.87 \|  \| 2.89 \|  \| 2.83 \|  \| 2.08 \|  \| \| Median \|  \| 3 \|  \| 3.00 \|  \| 3 \|  \| 3.00 \|  \| 3 \|  \| 3.00 \|  \| 3 \|  \| 3 \|  \| 2 \|  \| \| IQR \|  \| 0.00 \|  \| 0.00 \|  \| 0.00 \|  \| 0.00 \|  \| 0.00 \|  \| 0.00 \|  \| 0.00 \|  \| 1.00 \|  \| 2.00 \|  \| \| Shapiro-Wilk W \|  \| 0.643 \|  \| 0.730 \|  \| 0.745 \|  \| 0.673 \|  \| 0.694 \|  \| 0.780 \|  \| 0.791 \|  \| 0.437 \|  \| 0.832 \|  \| \| Shapiro-Wilk p \|  \| < .001 \|  \| < .001 \|  \| < .001 \|  \| < .001 \|  \| < .001 \|  \| < .001 \|  \| < .001 \|  \| < .001 \|  \| < .001 \|  \| \|  \| \| \| \| \| \| \| \| \| \| \| \| \| \| \| \| \| \| \| \| | | | | | | | |
